# Obesity, Diabetes and COVID-19: An Infectious Disease Spreading From the East Collides With the Consequences of an Unhealthy Western Lifestyle

**DOI:** 10.3389/fendo.2020.582870

**Published:** 2020-09-17

**Authors:** Jeff M. P. Holly, Kalina Biernacka, Nick Maskell, Claire M. Perks

**Affiliations:** Faculty of Medicine, School of Translational Health Science, Southmead Hospital, University of Bristol, Bristol, United Kingdom

**Keywords:** diabetes, obesity, COVID−19, SARS-CoV-2, pandemic (COVID-19)

## Abstract

The pandemic of COVID-19, caused by the coronavirus, SARS-CoV-2, has had a global impact not seen for an infectious disease for over a century. This acute pandemic has spread from the East and has been overlaid onto a slow pandemic of metabolic diseases of obesity and diabetes consequent from the increasing adoption of a Western-lifestyle characterized by excess calorie consumption with limited physical activity. It has become clear that these conditions predispose individuals to a more severe COVID-19 with increased morbidity and mortality. There are many features of diabetes and obesity that may accentuate the clinical response to SARS-CoV-2 infection: including an impaired immune response, an atherothrombotic state, accumulation of advanced glycation end products and a chronic inflammatory state. These could prime an exaggerated cytokine response to viral infection, predisposing to the cytokine storm that triggers progression to septic shock, acute respiratory distress syndrome, and multi-organ failure. Infection leads to an inflammatory response and tissue damage resulting in increased metabolic activity and an associated increase in the mechanisms by which cells ingest and degrade tissue debris and foreign materials. It is becoming clear that viruses have acquired an ability to exploit these mechanisms to invade cells and facilitate their own life-cycle. In obesity and diabetes these mechanisms are chronically activated due to the deteriorating metabolic state and this may provide an increased opportunity for a more profound and sustained viral infection.

The current global pandemic is the third epidemic of a major severe acute respiratory syndrome (SARS) caused by a coronavirus this century. The initial SARS epidemic in 2002 affected 29 countries, primarily in the far east, with 8,098 cases and 774 fatalities (1). This was followed 10 years later by the Middle-East Respiratory Syndrome (MERS) in 2012 that affected 27 countries, mainly in the middle-east, with 2,494 cases and 858 deaths (1). Seven years later another coronavirus, SARS-CoV-2, closely resembling SARS-CoV-1, originated in China in late 2019 but has since spread to 213 countries and as of early August 2020 there have been over 20 million cases worldwide with three-quarters of a million deaths (www.worldometers.info/coronavirus/). As such this is the first coronavirus to have a major impact on Western countries. In contrast to many places in Asia, Africa, and South America, over the last century most infectious diseases have become well-controlled in the West. However, growing epidemics of chronic diseases linked to lifestyle, such as cardiovascular disease, obesity and diabetes, that first became apparent in the West, have now spread to other regions of the world that have adopted a Western lifestyle and have slowly become global pandemics. The obesity and diabetes epidemics are also now prevalent in the Middle East and in the East; a cross-sectional survey of 170,287 participants in China in 2013 estimated that 47% of the adult population had either diabetes or prediabetes (2), The SARS-CoV-2 is the first acute pandemic in which an infectious disease from the East has collided with the slow pandemic of chronic lifestyle-related conditions from the West with severe consequences.

In contrast to the previous coronavirus epidemics, SARS-CoV-2 has spread more widely because its transmission has been facilitated by being highly contagious, in combination with a long latency period and large numbers of asymptomatic carriers (3). The SARS-CoV-2 virus results in a disease, COVID-19, that in some individuals can progress from a mild respiratory infection to a generalized inflammatory state, acute respiratory distress syndrome (ARDS) and ultimately multi-organ failure (MOF) associated with a high mortality rate (4). There are now seven coronaviruses that have spread to humans with HCov-229E, HCoV-OC43, HCoV-NL63, and HCoV-HKU1 thought to be responsible for around 30% of cases of the common cold (5). In around 80% of individuals, infection with SARS-CoV-2 can similarly result in mild or no discernible symptoms, but in around 20% of those infected COVID-19 can progress to severe outcomes with a high risk of mortality. The risk of severe disease is greatly enhanced by co-morbidities; the most common co-morbidities conferring the greatest risk are the chronic conditions associated with a Western-lifestyle; cardiovascular disease, obesity, and diabetes (6). A Western lifestyle is characterized by increased consumption of energy-dense foods and reduced physical activity leading to metabolic dysregulation with the global incidence of diabetes estimated to be some 463 million people in 2019 (7). The enforced stay-at-home/shield-in-place orders, that became almost universal, potentially resulted in reduced physical activity, altered nutritional intake and increased stress levels; all factors that could aggravate these conditions. In addition, reduced sun-light exposure and consequently reduced Vitamin D, reducing its anti-inflammatory actions (8) could also potentiate insulin-resistance (9). Indeed, using a simulation model that was created using glycemic data measured during previous disasters in India, with a similar impact to the current lockdown, it was predicted that after 45 days of lockdown glycemic control would deteriorate in patients with diabetes with a 3.68% increase in HbA1c (10).

## Obesity, Diabetes Effects on COVID-19 Risk, and Severity

That the metabolic disorder of diabetes was associated with COVID-19 was reported during the original outbreak in China and as the epidemic spread to Italy and the USA this was confirmed and similar associations were then also observed with obesity (11) A meta-analysis of 6 studies from China, that included 1,527 confirmed cases with SARS-CoV-2 infections, found a prevalence of diabetes among the patients of 9.7% compared to a prevalence within the general Chinese population of 10.9% (12) and an audit of 146 patients hospitalized with COVID-19 in Padova in Italy observed a prevalence of 8.9% (95% CI 5.3–14.6) compared to a prevalence of 11.0% in the same region (13). These preliminary reports would suggest that subjects with diabetes have no increased susceptibility to being infected; however, such conclusions are premature as there are many confounding issues that are yet to be examined including patients with obesity or diabetes being more likely to stay at home during such epidemics, being less mobile, have fewer social contacts and hence may have less exposure to the virus. Furthermore, the question of whether pre-existing diabetes or obesity predispose to acquiring an infection with SARS-CoV-2 is not possible to assess from reports of incident cases that depend considerably on the existing testing regimes which have varied greatly as the pandemic has developed and across different countries. If these conditions increased the severity of symptoms then there could be ascertainment bias in testing and detection of cases. Whether these preconditions predisposed to infection will only be answered when serological studies, monitoring the presence of viral antibodies in populations, have been completed with appropriate consideration for all confounding issues.

In contrast to the uncertainty regarding potential effects on the risk of infection, it is now clear that pre-existing diabetes and/or obesity have profound effects on the subsequent course of disease with many consistent reports of strong associations with morbidity and mortality. In the original outbreak in Wuhan it was observed that diabetes was associated with a higher risk of severe pneumonia, release of tissue injury-related enzymes, excessive uncontrolled inflammatory responses, and a hypercoagulable state (14). A report from the Center for Disease Control and Prevention in China, summarizing findings from 72,314 cases, observed that the overall case fatality rate was 2.3%, but for cases with diabetes the fatality rate was 7.3% (15). A review of hospital records of 1,099 patients in China found that the overall prevalence of diabetes was 7.4%; however, in those who required intensive care or mechanical ventilation or who died the prevalence was 26.9% compared to a prevalence of 6.1% in those without these indications of severe disease (16). A similar review of patient charts in Italy found that of 355 deaths 35.5% had pre-existing diabetes (6). A review of 5,279 confirmed infections at a single Medical Center in New York observed that overall 22.6% had pre-existing diabetes, but in those not requiring hospital admission only 9.7% had diabetes compared to 34.7% of those admitted (17). An initial assessment of co-morbidities among affected cases in the USA by the Center for Disease Control found that of 7,162 cases with full records overall 10.9% had diabetes but the prevalence was only 6% of those not requiring hospital admission compared to 24% of those requiring admission and 32% of those subsequently admitted to Intensive Care Units (ICU) (18). A study of 1,158 patients admitted to hospital in Kuwait, of which 104 needed ICU care, found the prevalence of diabetes to be 23.4% and in a multivariate analysis diabetes increased the risk of need for subsequent ICU with an OR 5.49 (CI 3.13, 9.65) (19).

There have also now been several meta-analyses of the many studies documenting the effects of diabetes and obesity on the severity and outcomes of COVID-19. A meta-analysis including data from 31 studies with a total of 6,104 cases found that cases with pre-existing diabetes had an OR of 2.61 (CI 2.05, 3.33) for developing severe COVID-19 compared to cases without diabetes (20). Another meta-analysis of 14 studies including 4,659 cases from China and USA with a prevalence of diabetes of 23.8% found that pre-existing diabetes increased the risk of death with OR 2.0 (CI 1.7, 2.3) (21). A larger meta-analysis including 33 studies with 16,003 cases found a prevalence of diabetes overall of 11.2%, but sub-group analysis revealed a prevalence of 10.5% of cases in China and 19.3% of cases outside of China (mainly USA): diabetes increased the risk of severe disease with OR 2.75 (CI 2.09, 3.62) and death with OR 1.90 (CI 1.37, 2.64) (22). A further meta-analysis of 30 studies with 6,452 cases found that diabetes increased the risk of severe COVID-19 with OR 2.45 (CI 1.79, 3.35), of ARDS with OR 4.64 (CI 1.68, 11.58) and of death with OR 2.12 (CI 1.44, 3.11) (23). When all of these outcomes were combined in an analysis of composite poor outcome, diabetes increased the risk with OR 2.38 (CI 1.88, 3.03) and a subgroup analysis revealed the risk was stronger in the younger cases with median age <55 years-old (RR 3.48) compared to older cases ≥55 years-old (RR 1.92) (23).

Associations between obesity and severity of COVID-19 or mortality have similarly been consistently reported. An audit of 124 patients admitted to ICU in France observed that among patients with COVID-19, obesity (BMI >30– <35 kg/m^2^) and severe obesity (BMI >35 kg/m^2^) were more prevalent than in control patients admitted to ICU for other causes: 47.6 vs. 25.2% and 28.2 vs. 10.8%, respectively, and those requiring invasive mechanical ventilation were also more obese (24). In a multivariate analysis of hospitalized patients in Kuwait, the risk of requirement for ICU was increased in the obese OR 2.7 (CI 1.17, 6.20) and those with morbid obesity (BMI >40) OR 3.95 (CI 1.0–15.2) (19). A similar study from New York that included 1,331 hospitalized COVID-19 patients, of whom 431 were admitted to ICU, found that the association with obesity was only evident in younger patients <60 years old with a higher risk of ICU admission for those with obesity (OR 1.8, CI 1.2, 2.7) and severe obesity (OR 3.6, CI 2.5, 5.3) compared to patients with a BMI <30 (25). An observational study of 20,133 patients admitted to 208 hospitals across the UK found that obesity increased the risk of death OR 1.33 (CI 1.19, 1.49) (26).

These observations are consistent with the long-known effects of obesity and diabetes on the severity and prognosis of infections. Influenza is often more severe and more often results in pneumonia in subjects with diabetes (27, 28). Following the influenza pandemic due to the H1N1 virus in 2009 obesity was recognized as a risk factor for hospitalization, the need for mechanical ventilation and death (29, 30). Plasma glucose and pre-existing diabetes were reported to be independent risk factors for morbidity and mortality in the original SARS epidemic in 2002 (31) and diabetes was strongly associated with mortality in the MERS epidemic in 2012 (32).

## Obesity, Diabetes Affect the Host Response to SARS-CoV-2

The metabolic derangements associated with obesity and diabetes have multiple effects on how the body responds to viral infections that could impact on the course of the disease. The most fundamental effect, that underpins the generalized increased predisposition to all infections, is the compromised immune system that is secondary to the metabolic derangements in obesity and diabetes. Interestingly, just a few years ago, as the health problems associated with a Western lifestyle spread to tropical regions, it was prophesized that this would pose a large threat for subjects with diabetes who contracted infectious diseases (33). With SARS-CoV-2 this threat is no longer confined geographically but has now become a global reality. Many aspects of the innate and adaptive immune systems are impaired in diabetes and obesity including inappropriate T-cell action, impaired natural killer cell activity, phagocytic cell dysfunction, inhibition of neutrophil chemotaxis, and defects in complement action (34–37). The compromised immune system in subjects with diabetes results in impaired responses to many of the stimuli activated during infections (38). Another potential consequence of the impaired immune cell function is that viral clearance could be reduced. A recent report measuring the interval between hospital admission and two negative tests for SARS-CoV-2 RNA, at least a day apart, found some evidence that there was delayed viral clearance in patients with diabetes (39). In addition, they found evidence that the use of glucocorticoids could also delay viral clearance (39) which is significant as glucocorticoids are frequently used for patients with ARDS (40). Indeed, glucocorticoid use is known to impair glycaemic control (41) and has been reported to reduce angiotensin-(1-7)-Mas receptor expression (42). However, although glucocorticoid use has been advised against in patients with COVID-19 (43) this advice has been questioned with a suggestion that low-to-moderate dose glucocorticoid could still be beneficial in treating critically ill patients with COVID-19 (44). Critical illness is associated with corticosteroid insufficiency (45) and there are cogent arguments for the use of glucocorticoids in critically ill patients (46) especially for treating ARDS (47). Consistent with this glucocorticoid treatment was found to reduce mortality in critically ill patients with SARS (48). A recent report confirmed similar findings in patients with COVID-19: in 2,014 patients randomized to receive dexamethasone, 28-day mortality was unaffected in subjects receiving no respiratory support but was reduced in critically ill patients receiving oxygen without invasive mechanical ventilation and was even more reduced in those receiving invasive mechanical ventilation (mortality rate ratio, 0.64; 95% CI, 0.51–0.81) (49). The compromised immune system in subjects with diabetes also increases susceptibility to potential secondary bacterial infection in the lungs (50).

Both obesity and type 2 diabetes are associated with chronic low-grade inflammation. Excess calorie intake results in a stimulation of pancreatic β-cell insulin secretion with the increase in oxygen consumption resulting in cell stress and mild inflammation. The insulin promotes glucose uptake and enlargement of adipocytes that in turn causes activation and recruitment of resident macrophages in adipose tissue. The adipocytes and macrophages then release more of a variety of proinflammatory cytokines and chemokines (including interleukin-1 (IL-1), IL-6, IL-8, monocyte chemoattractant protein-1 (MCP-1), C-reactive protein (CRP)) and less anti-inflammatory cytokines and adipokines (including IL-4, IL-10, IL-13, and adiponectin) (51, 52). These factors can then aggravate insulin-resistance leading to increased pancreatic insulin release and establish a vicious cycle. In subjects with obesity and diabetes the chronic low-grade inflammatory state could then aggravate the inflammatory response to SARS-CoV-2 infection and precipitate the hypersensitivity state and cytokine storm that can lead to pneumonia, ARDS and ultimately MOF observed in severe COVID-19 cases (16, 53, 54). Consistent with this, higher levels of IL-6, CRP and fibrinogen were observed in COVID-19 patients with diabetes compared to those without (14). The cytokine storm is part of an evolutionary conserved stress response preparing the body for a severe insult (55) with activation of the hypothalamic-pituitary-adrenal (HPA) axis (to produce cortisol), the sympathetic nervous system (to generate catecholamines), a tissue defense response and an acute-phase reaction, to generate pro-coagulant factors, in preparation for tissue damage.

Insulin-resistance, obesity, and diabetes promote an atherothrombotic state due to a disturbance in the balance of factors regulating coagulation and fibrinolysis with an increase in many clotting factors (such as tissue factor and fibrinogen) and adhesion molecules (such as P-selectin), a reduction in anticoagulant proteins (such as antithrombin) and a reduction in fibrinolysis due to an increase in plasminogen activator inhibitor type-1 (PAI-1) (56, 57). These factors could increase the likelihood of the development of endothelial dysfunction and platelet aggregation promoting the formation of occlusive thrombus in the heart and lungs in COVID-19 patients with diabetes. In patients with COVID-19 however there are increased levels of fibrinogen, CRP, and D-dimer (14, 58), with elevated D-dimer levels being a risk factor for mortality (53) these findings indicate that there appears to be an increase not only in coagulation but also in fibrinolysis. An imbalance or an impairment of coordination between coagulation and fibrinolysis appears to be responsible for much of the pathology observed in COVID-19 (59). The dysregulation of the system in subjects with obesity and diabetes could then exacerbate the effects of COVID-19 (60). Indeed, lung, heart, and brain damage appear to be common pathological findings in COVID-19 associated with fibrotic clots and disseminated intravascular coagulation (61, 62).

Severe obesity is associated with restrictive lung function with decreased expiratory reserve volume, functional capacity, and respiratory system compliance. These factors can complicate the clinical management of obese COVID-19 patients and in subjects with excess abdominal adipose tissue the diaphragmatic excursions are compromised and this can make assisted ventilation more problematic (63).

Diabetes and hyperglycaemia are accompanied by an accumulation of advanced glycation end-products (AGEs) that interact with a specific cell surface protein, the receptor for AGEs (RAGE). This has subsequently been found to be a pattern recognition receptor (PRR) that also recognizes pathogen-associated molecular patterns (PAMPs) from microorganisms, as well as danger-associated molecular patterns (DAMPs) released by stressed or damaged cells and play a key role in the inflammatory response (64). Although RAGE is normally expressed at low levels in most tissues and increased during inflammation, it is expressed at high levels in Type I and Type II alveolar epithelial cells (AT1, AT2) in the lung and appears to be a critical mediator of the pulmonary inflammatory response (65). In addition, it has been shown that activation of the Angiotensin II receptor 1 (AT1R) by Angiotensin II (AngII) can transactivate RAGE and that this mediates the subsequent inflammatory response (66). It has been suggested that RAGE may aggravate the inflammation and coagulation observed in COVID-19 (67) and contributes to the lung pathology (68). Infection with SARS-CoV-2 perturbs the renin–angiotensin system favoring AT1R activation (see below) and hence potentially transactivating RAGE, which is also activated by accumulated AGEs in patients with diabetes, aggravating the inflammatory and fibrotic response and promoting lung damage.

In addition to the general effect of inflammatory cytokines aggravating insulin-resistance, it had previously been reported that the SARS-CoV-1 receptor, which is the same receptor used by SARS-CoV-2, was abundantly expressed in the pancreas and that a proportion of patients with SARS developed transient diabetes during the course of the disease (69). This suggests that infection with SARS-CoV-2 could lead to a deterioration in metabolic control via a number of mechanisms and the metabolic derangements could, in turn, promote a more severe disease; this could be a particular problem in patients with obesity and those with type 2 diabetes who have residual pancreatic function in whom metabolic perturbations were already present.

## Obesity, Diabetes, and the Viral Pathway

**Figure 1:**
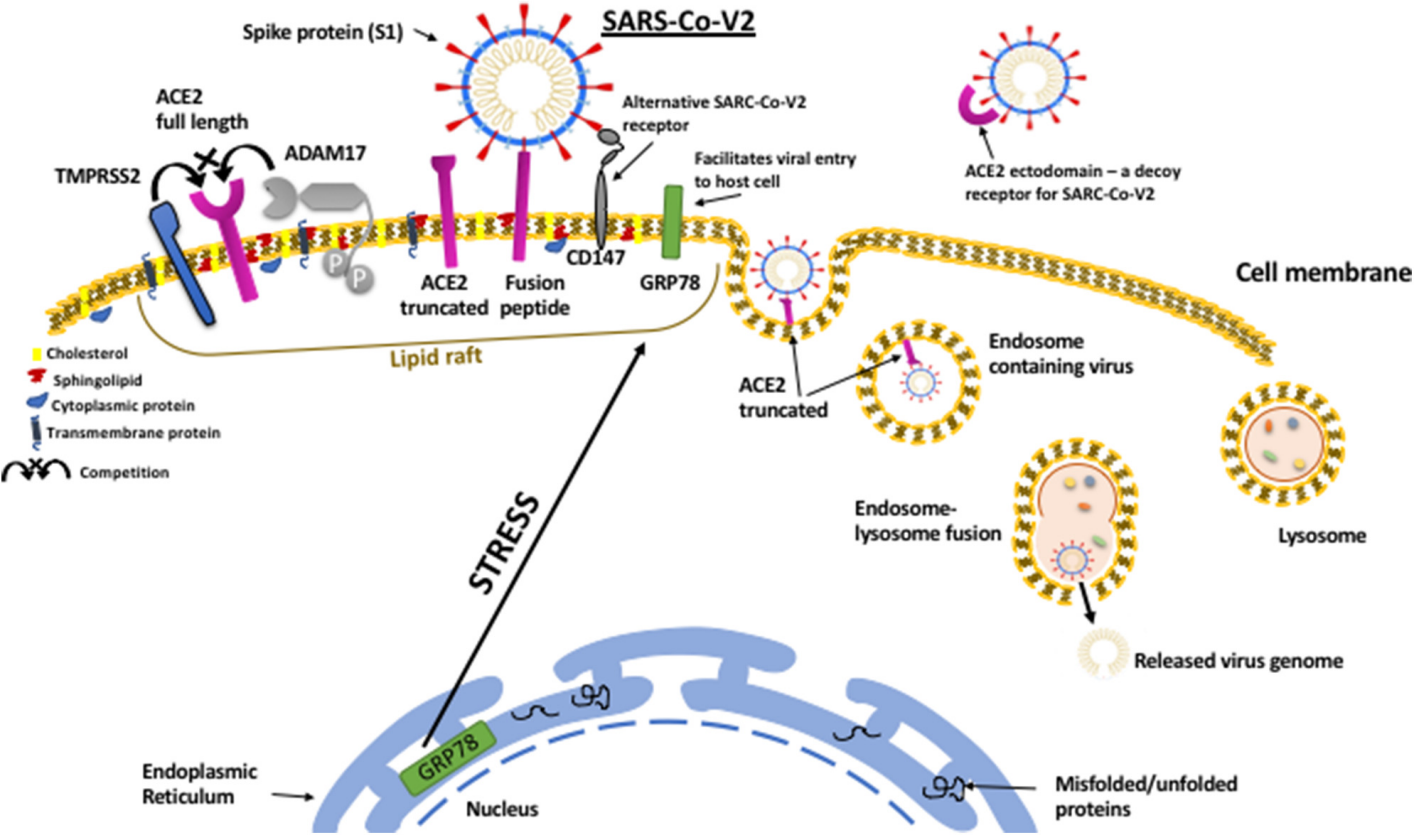
SARS-CoV-2 viral entry to host cell pathway. The SARS-CoV-2 virus attaches to host cell surfaces via specific receptors, ACE2 and CD147, via spike protein (S1) projecting from the viral envelope. The spike protein first has to be “primed” by proteolytic cleavage by TMPRRS2 which can also cleave ACE2 resulting in shedding of the ectodomain. In contrast to TMPRSS2, which facilitates virus binding, ACE2 can also be cleaved by ADAM17, which prevents viral binding and results in shedding of the ACE2 ectodomain that can still bind to SARS-CoV-2 and act as a decoy receptor reducing viral infection. The entry of the virus is facilitated by the protein chaperone, GRP78 that also binds to the spike protein. Metabolic stress in the host cell results in upregulation of GRP78 and its translocation to the cell surface. On the cell surface ACE2, CD147, GRP78, TMPRSS2, and ADAM17 all cluster within organized cholesterol-rich domains called lipid rafts and together can enable viral entry via endocytosis. Viral replication within the cell can also be facilitated by GRP78.

In addition to the many established mechanisms whereby metabolic disorders could promote a more severe clinical response to SARS-CoV-2; the emerging evidence, uncovering how the virus infects cells, has indicated many further links to endocrine and metabolic controls. This has raised the prospect that obesity and diabetes could promote the course of the viral infection within the body. In order to enter and infect host cells the virus appears to have hijacked mechanisms that have evolved to ingest and degrade foreign material; with SARS-CoV viral entry occurring mainly by endocytosis via the endosomal/lysosomal route (70)(Figure 1).

### Angiotensin Converting Enzyme 2 (ACE2)

The initial attachment of SARS-CoV-2 virus to cells has been reported to be mediated by ACE2, a transmembrane protein known to be important in the renin-angiotensin system (RAS) (71, 72). There are two immediate implications: the pattern of expression of ACE2 may determine the tissues in the body that are most affected by infection and secondly the virus may perturb the normal function of RAS which could impact the course of the disease. Two forms of ACE2 are found in the body, the full-length transmembrane form and a soluble form generated by proteolytic shedding of the extracellular domain of ACE2. The normal enzyme function of ACE2 is to degrade AngII to Ang-(1-7) and to a lesser extent AngI to Ang-(1-9); this in effect then opposes the action of ACE which converts Ang I to Ang II (73). The RAS plays an important role in regulating vascular function, blood pressure, and fluid and electrolyte balance. It comprises a dynamic counter-regulatory system with Ang II, the product of ACE, activating the AT1R to promote vasoconstriction, salt and water retention, fibrosis, and inflammation whereas the product of ACE2, Ang-(1-7) activates the G protein-coupled receptor Mas-receptor (MasR) to stimulate vasodilation and anti-inflammatory effects. The expression of ACE2 has been described in a number of tissues including the heart, kidneys, pancreas, and both type I and type II alveolar epithelial cells (ATI, ATII) in the lungs (74, 75). In addition to its well-known role in the cardiovascular system, the RAS also plays a critical role in lung function and disturbances to the ACE/ACE2 balance can lead to pulmonary disease. Knock-out of ACE2 in mice results in severe ARDS (76). In addition, ACE2 was shown to play an important role in lung injury in mice infected with H5N1 (77) and also infected with SARS-CoV-1 (78).

It has also become increasingly clear that ACE2 may play an important role in metabolic regulation. Knock-out of the ACE2 gene in mice resulted in a deficiency of pancreatic insulin secretion that was partially countered by increased glucose utilization in muscle (79, 80). Knock-down of ACE2 also compromises pancreatic function in obese mice (81). In an experimental model in which a high-calorie diet induces insulin resistance in mice, knock-down of ACE2 exaggerates the insulin resistance by reducing glucose uptake into tissues (82). Furthermore, overexpression of ACE2 improved glycaemic control in a model of type II diabetes in mice (83). In addition to SARS-CoV-2 directly infecting the pancreas via ACE2 and causing transient diabetes (69) the interaction of the virus with ACE2 could therefore have other effects to perturb the RAS and further compromise metabolic control.

The levels of ACE2 have been reported to be decreased in some experimental models of diabetes (84) and the loss of the counter-regulatory protective effects of the RAS has been speculated to potentiate lung damage. However, other reports indicate increased levels of ACE2 in the heart, liver, and lungs of mice with diabetes (85) and it has been suggested that this could make these tissues more vulnerable to SARS-CoV-2 infection and contribute to an increased risk of MOF in patients with diabetes. Evidence from humans indicate that levels of ACE2 in urine are increased in patients with type I diabetes (86) and type II diabetes (87) and positively related to blood glucose and HbA1c levels (88). It is not yet clear however, whether increased ACE2 in the urine is due to increased shedding of ACE2 in the kidney or whether it reflects increased circulating or systemic tissue levels. However, increased serum levels of ACE2 have been reported in subjects with diabetes and obesity (89). Expression of ACE2 in sputum cells has also been reported to be increased in subjects with diabetes and to be decreased in subjects using inhaled corticosteroids (90). Furthermore, a Mendelian Randomization study indicated that diabetes was causally related to increased ACE2 expression in lung tissue (91). An increase in ACE2 expression was also recently reported in the kidney of patients with diabetic kidney disease (92) and in the liver of patients with diabetes and also subjects with non-alcoholic fatty liver, which is prevalent in subjects with prediabetes (93). The weight of evidence therefore suggests that ACE2 levels are probably increased in various tissues in humans with diabetes and as evidence from cell biology suggests this may increase viral entry, this implies that infection with SARS-CoV-2 may be increased in tissues such as the lung, liver and kidney.

Following binding of SARS-CoV to ACE2, the complex is endocytosed and proteolyzed resulting in reduced tissue levels of ACE2 (94–96). Consistent with a down-regulation of ACE2, increased levels of AngII have been observed in patients with COVID-19 and these correlated with viral load (97). The increased levels of Ang II, together with potentially reduced levels of Ang-(1-7), would shift the balance of RAS actions to pro-inflammatory, rather than anti-inflammatory, resulting in increased lung damage as has been shown in experimental models of SARS (76, 78). However, it has recently been reported that expression of ACE2 can be upregulated by inflammatory cytokines (98) and this has also been reported to occur in airway epithelium (99). The chronic inflammatory state found in diabetes and obesity could facilitate viral infection via such a mechanism. In addition, insulin decreases expression of ACE2 (85, 100) suggesting that metabolic regulators could play an important role in affecting viral entry and/or in altering the inflammatory response by shifting the balance in the RAS. It has also been reported that the virus itself could upregulate ACE2 expression suggesting a positive feed-forward loop to enhance infection (101).

## The Type II Transmembrane Serine Protease (TMPRRS2)

The virus interacts with ACE2 via a specific spike protein (S-protein) projecting from its envelope. The S-proteins of SARS-CoV viruses are typical class I viral fusion proteins that first need to be cleaved by proteases to activate their fusion ability that enables the virus to invade the host cell (70). The S protein is cleaved into two subunits (S1 and S2); the S1 subunit is then further divided into SA and SB domains, with the SB domain predicted to bind to human ACE2. This is often referred to as S-protein priming. The S2 subunit is responsible for fusion of the virus–ACE2 complex with the cell membrane. The enzyme responsible for S-protein priming and SARS-CoV-2 entry has been identified as TMPRSS2 and a clinically approved TMPRSS2 inhibitor, Camostat, markedly reduced viral entry into cultured lung cells (102). Similar results were reported previously for SARS-CoV-1 cell entry (103).

Expression of TMPRSS2 has been described in epithelial cells across a variety of tissues including prostate, colon, small intestine, pancreas, kidney, liver, and lung (104). It has been most studied in the prostate where it is strongly upregulated by androgens via an androgen-responsive promoter enhancer in the TMPRSS2 gene (105). Interestingly, it has also been reported to be upregulated on lung cells by both androgens and glucocorticoids (106). The strong androgen dependence of TMPRSS2 could contribute to the increased risk for men with COVID-19 (107). In addition to cleaving the S-protein, TMPRSS2 also cleaves ACE2 resulting in shedding of its ectodomain creating a soluble form of ACE2. This then competes with the normal shedding of ACE2 due to cleavage by the cell surface protease a disintegrin and metallopeptidase domain 17 (ADAM17) also known as tumor necrosis factor-converting enzyme (TACE) (108). Cleavage and shedding of ACE2 by ADAM17 prevents the action of TMPRSS2 to facilitate viral entry and in addition the soluble shed ACE2 can still bind to the viral S-protein and hence can compete with cell surface binding and protect tissues from infection acting as a decoy receptor (103). Although, as described above, a loss of the counter-regulatory protective effects of the RAS may enhance the damage in already infected lungs.

Whether TMPRSS2 has a role in metabolic regulation has yet to be investigated. The TMPRSS2 inhibitor, Camostat, has been reported to correct some of the metabolic abnormalities present in rats with diabetes and obesity (109), however, it is not clear that these effects were specific to inhibition of TMPRSS2.

## Lipid Rafts

Viral entry depends on an interaction between the virus S-protein and both ACE2 and TMPRSS2 on host cell surfaces and this occurs within organized domains called lipid rafts that are rich in cholesterol and sphingolipids; agents that disrupt lipid rafts prevent SARS-CoV-2 viral entry (110, 111). Lipid rafts were previously shown to play a role in SARS-CoV-1 infection (112), specifically that the interaction of SARS-CoV-1 S-protein with ACE2 occurred within lipid rafts (113). Indeed, lipid rafts have previously been implicated as important cell surface domains for the entry of other viruses (114, 115). An increase in cholesterol increases the availability of SARS-CoV-2 viral entry points (111). As cholesterol levels increase with aging and with metabolic disorders this could contribute to their associations with the severity of COVID-19. In addition, 7-ketocholesterol (7-KC) and 25-hydroxycholesterol (25-HC; a major circulating metabolite of cholesterol), can replace cholesterol in lipid rafts, disrupting their organization and these oxysteroids have been reported to have anti-viral activity (116–119) including against a porcine coronavirus (120). Inflammatory cytokines stimulate the formation of 25-HC, which in turn, promotes adipose tissue inflammation that is found in diabetes and obesity (121). The targeting of cholesterol and lipid metabolism and distribution has been proposed as a novel strategy for treating viral infections (122). The need for clustering of ACE2 and TMPRSS2 within organized domains in lipid rafts for viral entry could help explain some of the seemingly discrepant reports that increased ACE2 could facilitate infection, as described above, or actually protect against infection (123). With ACE2 present as a soluble, shed form that could act as a decoy-receptor and on cell surfaces in raft and non-raft domains, the total abundance may be less important than the localization and distribution of ACE2.

## CD147

In addition to the virus gaining entry to host cells via an interaction of the viral S-protein with ACE2, viral entry has also been reported to occur via an interaction between the S-protein and CD147, with viral entry blocked by an antibody to CD147 (124). Again, this route had previously been reported for SARS-CoV-1 (125) and for other viruses including hepatitis B, human cytomegalovirus, Kaposi's sarcoma–associated herpesvirus, measles and HIV-1 (126–128). In turn, viral infection of host cells leads to upregulation of CD147 (129).

CD147, which is also known as Basigin or extracellular matrix metalloproteinase inducer (EMMPRIN), is a transmembrane glycoprotein that belongs to the immunoglobulin superfamily (126). CD147 is expressed in a number of cells in the lungs and its expression is increased in patients with chronic obstructive pulmonary disease (COPD) (130) and at sites of lung fibrosis (131). The expression of CD147 on the cell surface is upregulated by high glucose levels and by AGEs, via activation of RAGE, and has been suggested to play a role in diabetic complications (132). On cell surfaces CD147 is found within lipid rafts associated with caveolin-1 (133). In addition, HMG-CoA reductase inhibitors, statins, inhibit the cholesterol biosynthesis pathway and disrupt lipid raft composition resulting in a reduction of CD147 translocation to the cell surface (134). In addition to playing a role in viral entry, CD147 has a role in the immune response by modulating T-cell activation (135). One of the other prime functions of CD147 appears to be as a chaperone for monocarboxylate transporters (MCTs) that facilitate the transport of monocarboxylates such as lactate across the plasma membrane (136). In addition, CD147 is colocalized and associates with the glucose transporter, GLUT1, on cell surfaces (137) and an increase in CD147 is accompanied by an increase in GLUT1 and a switch to more glycolytic metabolism (138). Therefore, CD147 appears to be an important determinant of cell metabolism and in addition to facilitating SARS-CoV-2 entry and modulating the immune response, the virus interaction with CD147 could affect cell metabolism.

## Glucose-Regulated Protein 78 (GRP78)

A further important component in viral entry appears to be GRP78. Molecular modeling predicts that the SARS-CoV-2 S-protein should bind to GRP78 with high affinity (139). This would be consistent with a previous report that GRP78 bound to the S-protein of other coronavirus and was permissive for their entry to host cells, including MERS-CoV and HKU9 (140). The most well-established role of GRP78 is as a protein chaperone that ensures the correct folding and assembly of proteins in the endoplasmic reticulum (ER) and in the event of accumulation of misfolded proteins aids their degradation or initiates the unfolded protein response (UPR) or the ER stress response (141, 142). The chaperone role of GRP78 acts not only in the ER but also to chaperone proteins entering the cell via endocytosis (143). As viral proteins are foreign to host cells, viruses that acquired an ability to engage with host cell chaperones and disrupt the process designed to degrade unrecognized proteins would have gained a clear advantage. As such GRP78 binding appears to be have been commonly acquired by many viruses to not only ensure safe entry to host cells but also to facilitate viral replication. When a cell starts to make new viral proteins, it is an advantage for the virus if these are not immediately degraded. Infection with Coxsackievirus A9 (CAV-9) causes clustering of GRP78 with integrin receptors within lipid rafts on host cell surfaces which together then facilitate viral entry (144). The endocytosis of Zika virus is also facilitated by cell surface GRP78 (145). Similarly, GRP78 acts as a receptor for Dengue virus and antibodies to GRP78 could inhibit infection (146). Denge virus induces increased expression of GRP78 in host cells which then acts as a chaperone to facilitate viral protein production and viral replication (147). Similarly, Japanese Encephalitis Virus not only employs GRP78 as a receptor for host cell entry but also in facilitating viral replication (148). Although binding to GRP78 appeared not to be required for the entry of Ebola virus into host cells, it did play an essential role in viral protein transcription (149).

While GRP78 belongs to the heat shock protein 70 (HSP70) family, rather than being induced by heat shock it is induced by metabolic stress and was named due to observations of its induction by glycaemic stress (142, 150). The significance of the metabolic regulation of GRP78 is still far from being fully understood. However, it is clear that metabolic disturbances can have profound effects on the expression and functions of GRP78. Cell surface GRP78 can be shed and circulating GRP78 levels were found to be increased in subjects with diabetes and obesity and to correlate with CRP levels, suggesting that the chronic inflammation could be a contributing factor to the observed increase in addition to the metabolic stress (151). Indeed, as well as induction by metabolic stress, inflammatory cytokines also appear to stimulate the translocation of GRP78 to cell surfaces (152). Accumulation of AGEs can also induce GRP78 via activation of RAGE (153, 154). In a cell model of diabetic nephropathy high glucose was shown to upregulate cell surface GRP78 where it associated with integrin receptors and enhanced a fibrotic response (155). Androgens have also been reported to upregulate GRP78 (156, 157) and this could be a further contributing factor to the increased risk of COVID-19 in males. Other relevant functions of cell surface GRP78 are its ability to bind and stabilize ADAM17 (158) and also bind to tissue factor and regulate the initiation of coagulation (159).

The chaperone role of GRP78 acts not only in the ER but also to chaperone proteins entering the cell via endocytosis (143). It seems that viruses have commonly acquired an ability to bind to GRP78 and hijack its chaperone function to both facilitate entry to host cells and to enable the production and assembly of viral proteins. The role of GRP78 in SARS-CoV-2 infections has yet to be defined although in a clinical study serum levels of GRP78 were found to be elevated in patients admitted with COVID-19 compared to patients with pneumonia or healthy controls (160). The available evidence indicates that GRP78 is increased and is translocated to cell surfaces in patients with metabolic disorders and this could play an important role in viral entry to host cells and in viral replication, as well as contributing to the fibrotic and coagulation responses.

## Clinical Implications

There are a number of implications from these observations. Reducing excess weight gain and improving metabolic health, in addition to all of the obvious well-known health benefits, may help prevent against a severe COVID-19 response to infection. Special protection/shielding should be provided to those with pre-existing obesity and diabetes. However, in contrast to the implication that the androgen dependence of COVID-17 severity may offer a strategy for using counter-measures to protect against COVID-19 (161), the metabolic effect needs more careful consideration. The management of metabolic control in critically ill patients is much more challenging than in those not acutely ill and many of the normal drugs used in diabetes are counter-indicated, with insulin being the most appropriate therapy (162). In addition, there are good reasons why glucocorticoids, which may seem counter-indicated, may actually be very beneficial in critically ill patients (46). In addition, the intense focus on SARS-CoV-2 has led to many advances in our understanding of the viral pathway that could provide new targets for developing more effective therapies against this and other viruses. The use of statins may have several benefits: the disruption of lipid raft composition (134) could reduce the various pathways of viral entry (163). In addition, statins have general anti-coagulant and anti-inflammatory effects (163); the latter could be particularly beneficial in combination with glucocorticoids in critically ill patients (46).

## Summary

The consequences of adopting a lifestyle, consuming excess calories with limited physical activity, are the metabolic derangements culminating in diabetes and obesity that are now at pandemic levels throughout the West. It has become clear that these conditions predispose individuals to severe COVID-19 that is caused by a virus spreading from the East, that in the majority of cases causes mild influenza-like symptoms. There are many consequences of diabetes and obesity that may accentuate the clinical response to SARS-CoV-2 infection. These include an impaired immune response, an atherothrombotic state, accumulation of AGEs activating RAGE and especially the pre-existing chronic inflammatory state. The later could prime an exaggerated cytokine response to viral infection, predisposing to the cytokine storm that triggers progression to septic shock, ARDS, and MOF.

In addition, to all of these factors that may contribute to these metabolic conditions exacerbating the clinical course of COVID-19, there are more fundamental mechanisms that may contribute to facilitating the viral infection. Infection leads to an inflammatory response and tissue damage and this results in increased metabolic activity. This is associated with an increase in the mechanisms by which cells ingest and degrade tissue debris and foreign materials. It appears that viruses have acquired the ability to exploit these mechanisms to invade cells and facilitate their own life-cycle. In obesity and diabetes these mechanisms are chronically activated due to the perturbed metabolism and this may provide an increased opportunity for a more profound and sustained viral infection.

## Author Contributions

All authors listed have made a substantial, direct and intellectual contribution to the work, and approved it for publication.

## Conflict of Interest

The authors declare that the research was conducted in the absence of any commercial or financial relationships that could be construed as a potential conflict of interest.
